# Region of origin and cervical cancer stage in multiethnic Hispanic/Latinx patients living in the United States

**DOI:** 10.1002/cam4.6697

**Published:** 2023-11-15

**Authors:** Andreea Ioana Dinicu, Shayan Dioun, Mandy Goldberg, Danielle M. Crookes, Yongzhe Wang, Ana I. Tergas

**Affiliations:** ^1^ Obstetrics, Gynecology and Women's Health Institute, Cleveland Clinic Cleveland Ohio USA; ^2^ Columbia University College of Physicians and Surgeons New York New York USA; ^3^ New York Presbyterian Hospital New York New York USA; ^4^ Joseph L. Mailman School of Public Health Columbia University New York New York USA; ^5^ Bouvé College of Health Sciences and College of Social Sciences and Humanities Northeastern University Boston Massachusetts USA; ^6^ Division of Gynecologic Oncology, Department of Surgery City of Hope Comprehensive Cancer Center Duarte California USA; ^7^ Division of Health Equity, Department of Population Science Beckman Research Institute, City of Hope Comprehensive Cancer Center Duarte California USA

**Keywords:** cervical cancer, health disparities, Hispanic, Latino/Latinx

## Abstract

**Background:**

Hispanic/Latinx people have the second highest cervical cancer incidence rates in the U.S. However, there is a lack of disaggregated data on clinical outcomes for this diverse and populous group, which is critical to direct resources and funding where they are most needed. This study assessed differences in stage at diagnosis of cervical cancer among Hispanic/Latinx subpopulations and associated factors.

**Methods:**

We analyzed patients with primary cervical cancer from 2004 to 2019 in the National Cancer Database. Hispanic/Latinx patients were further categorized into Mexican, Puerto Rican (PR), Cuban, Dominican, and Central/South American, as per standard NCDB categories, and evaluated based on stage at diagnosis and sociodemographic characteristics. Multinomial logistic regression quantified the odds of advanced stage at presentation. Regression models were adjusted for age, education, neighborhood income, insurance status, and additional factors.

**Results:**

Hispanic/Latinx cervical cancer patients were more likely to be uninsured (18.9% vs. 6.0%, *p* < 0.001) and more likely to live in low‐income neighborhoods (28.6% vs. 16.9%, *p* < 0.001) when compared to non‐Hispanic White populations. Uninsured Hispanic/Latinx patients had 37.0% higher odds of presenting with regional versus localized disease (OR 1.37; 95% CI, 1.19–1.58) and 47.0% higher odds of presenting with distant versus. Localized disease than insured patients (OR 1.47; 95% CI, 1.33–1.62). When adjusting for age, education, neighborhood income, and insurance status, PR patients were 48% more likely than Mexican patients to present with stage IV versus stage I disease (OR 1.48; 95% CI, 1.34–1.64).

**Conclusion:**

Disaggregating health data revealed differences in stage at cervical cancer presentation among Hispanic/Latinx subpopulations, with insurance status as a major predictor. Further work targeting structural factors, such as insurance status, within specific Hispanic/Latinx subpopulations is needed.

## 1 INTRODUCTION

In the U.S., nearly 14,000 individuals are diagnosed with cervical cancer, and over 4000 individuals die from it annually.^1^, ^2^ While overall incidence rates have declined by approximately 70% with implementation of routine screening with cytology in the mid‐1970's; trends vary by race and ethnicity. Hispanic/Latinx people have the second highest incidence rates of cervical cancer among any racial and ethnic group in the U.S., with rates of 9.7 per 100,000 as compared to non‐Hispanic White (NHW) populations who have rates of 7.2 per 100,000.^1^ Whereas rates continue to decline by 1%–2% annually for most groups in the U.S., rates for younger Hispanic/Latinx people increased by 2% per year from 2012 to 2019 according to the most recent data.^1^

The survival rate of people with cervical cancer is poor, with a 5‐year overall survival rate of 67%.^1^ Although patients with localized cervical cancer have a 5‐year survival rate of 92%, those with regional and distant disease have survival rates of 59% and 17%, respectively. One of the most significant factors that determines survival is stage at diagnosis.^3^ Hispanic/Latinx patients with cervical cancer are more likely to present with advanced‐stage cervical cancer compared to NHW individuals,^4^, ^5^, ^6^ and Hispanic/Latinx populations have experienced an increase in the proportion of cervical cancers diagnosed with distant disease.^5^ Socioeconomic inequities, as measured by variables such as median family income and poverty level, are thought to be a mediator of these findings.^4^ Other studies have shown that disparities in screening and insurance status are associated with diagnoses of advanced‐stage cervical cancer among Hispanic/Latinx populations.^6^, ^7^

The U.S. Hispanic/Latinx population is the fastest‐growing ethnic minority group and, by 2050, is expected to exceed a population of 102 million people from over a dozen countries of origin.^8^ Health data for Hispanic/Latinx populations are typically presented in aggregate, which masks the population's significant heterogeneity.^9^ However, there is evidence that important differences exist in cervical cancer outcomes between Hispanic/Latinx subpopulations. For example, incidence rates among people living in Puerto Rico are 30% higher than other U.S. Hispanic/Latinx groups. Additionally, prior work has shown higher cervical cancer mortality rates among Puerto Rican and Mexican populations when compared to patients of other Hispanic/Latinx descent.^8^, ^10^, ^11^ Despite the high incidence of cervical cancer among Hispanic/Latinx patients and the varying distribution of cancer mortality within subpopulations, there is a lack of disaggregated data on stage at diagnosis among different Hispanic/Latinx groups. Data disaggregation and specific characterization of Hispanic/Latinx subpopulations could contribute to the precision and reliability of research findings. Furthermore, it is integral for adequate resource allocation and development of community‐centered interventions and policies aimed at improving health equity.^12^ To address this, the current study examined patterns of stage at diagnosis of cervical cancer and associated factors among Hispanic/Latinx subpopulations living in the U.S.

## 2 METHODS

### 2.1 Data source

This study used the National Cancer Database (NCDB), a registry developed and sponsored by the American College of Surgeons and American Cancer Society.^13^ The NCDB captures data on all patients with newly diagnosed invasive cancers from over 1500 Commission on Cancer (CoC) affiliated hospitals across the United States. The NCDB collects data on patient characteristics, including cancer staging, tumor characteristics, first course of treatment, and outcomes. Incident tumor cases are compiled by trained registrars, and the data are audited regularly to confirm accuracy. Additionally, the NCBD contains demographic information, including race and ethnicity, and education and income level, which is abstracted from medical records or inferred according to patient zip code and Census data.^14^ This study was deemed exempt by the Columbia University Institutional Review Board due to the use of deidentified data.

### 2.2 Cohort selection

Patients aged 18 years and older with newly diagnosed cervical cancer from 2004 to 2019 were included in the analysis. Hispanic/Latinx individuals were further categorized by subgroups: Mexican, Puerto Rican (PR), Cuban, Dominican, and other Central/South American (CSA) as per standard NCDB categories. Inclusion criteria were based on primary invasive cervical cancer diagnosis in NHB, NHW, and Hispanic/Latinx patients. Exclusion criteria included unknown Hispanic/Latinx origin, nonprimary cervical cancer diagnosis, and having more than one reported cancer diagnosis. Patients with unknown stage at diagnosis were excluded from the regression analyses.

### 2.3 Outcomes and covariates

The primary dependent variable assessed was stage at diagnosis, categorized as a four‐level categorical variable (stage I, II, III, IV) for bivariate analyses and a three‐level categorical variable (localized: stage I, regional: stages II–III, distant: stage IV) for regression analysis as per the Surveillance, Epidemiology, and End Result (SEER) Database. Staging was classified using the 2009 International Federation of Gynecology and Obstetrics (FIGO) staging guidelines for cervical cancer.^15^ Education and income were measured at the local geographic level. Education level is coded in the NCBD as the percentage of adults without a high school diploma quartiles within the patient's zip code, ranging from <6.3% to >17.6% based on census data.^14^ Income was denoted as median income in the patient's zip code. Neighborhood income ranges were <$40,227, $40,227–$50,353, $50,354–$63,332, and ≥$63,333. Insurance status, defined by the NCDB as the patient's primary insurance carrier at the time of initial diagnosis and/or treatment, was categorized as not insured, privately insured, Medicaid, Medicare, other, and missing. Additional variables included tumor characteristics and year of diagnosis. Facility type was categorized as academic/research program (includes NCI‐designated comprehensive cancer centers), comprehensive community cancer program, community cancer program, integrated network cancer program, or other. Facility location was marked according to U.S. Census regions (Midwest, Northeast, South, and West).

### 2.4 Statistical analysis

Chi‐squared tests were used to detect differences in demographic and clinical characteristics between groups. In order to investigate the relationship between the Hispanic subpopulation and cervical cancer stages, multinomial logistic regressions were implemented, while adjusting for socioeconomic and clinical variables. The analysis consisted of three models. The crude model was only adjusted for age. The second model aimed to adjust for socioeconomic factors and included adjustments for age, education, neighborhood income, and insurance status. Finally, the third model aimed to adjust for both socioeconomic factors and clinical factors by accounting for age, education, neighborhood income, insurance status, tumor grade, tumor histology, Charlson–Deyo index, facility location, facility type, and year of diagnosis. Within all models, stage I for cervical cancer was selected as the reference level. The Mexican subpopulation was chosen as the reference level for all models because it was the largest group. All statistical tests were two‐tailed, and a significance level of *p* < 0.05 was used to determine statistical significance. The statistical analyses were performed using R version 4.2.1.

## 3 RESULTS

### 3.1 Patient demographics

Among 109,502 patients diagnosed with cervical cancer, 83,497 (76.2%) were NHW, 20,026 (18.3%) were NHB, and 5979 (5.5%) were Hispanic/Latinx (Table 1). Within the Hispanic/Latinx group, 3263 (54.6%) were Mexican, 615 (10.3%) PR, 261 (4.4%) Cuban, 229 (3.8%) Dominican, and 1611 (26.9%) CSA patients (Table 1).

**TABLE 1:** Patient demographics and malignancy‐related variables.

	Total (*N* = 109,502)	White, NH (*n* = 83,497; 76.2%)	Black, NH (*n* = 20,026; 18.3%)	All Hispanic (*N* = 5979; 5.5%)	*p* Value (Total)	Mexican (*n* = 3263; 54.6%)	Puerto Rican (*n* = 615; 10.3%)	Cuban (*n* = 261; 4.4%)	Dominican (*n* = 229; 3.8%)	South or Central America (*n* = 1611; 26.9%)	*p* Value (Hispanic only)
Age in years (mean, SD)	50.5 (14.5)	50.2 (14.5)	52 (14.9)	49.7 (14.3)	<0.001	49 (13.8)	50.4 (14.9)	54.1 (14.9)	54.5 (15.3)	49.3 (14.4)	<0.001
Education											
No HSD ≥ 17.6%	27,553 (25.2%)	15,514 (18.6%)	8464 (42.3%)	3575 (59.8%)	<0.001	2117 (64.9%)	309 (50.2%)	133 (51%)	144 (62.9%)	872 (54.1%)	<0.001
No HSD 10.9%–17.5%	28,389 (25.9%)	21,696 (26%)	5699 (28.5%)	994 (16.6%)		467 (14.3%)	142 (23.1%)	59 (22.6%)	36 (15.7%)	290 (18%)	
No HSD 6.3%–10.8%	24,855 (22.7%)	21,429 (25.7%)	2807 (14%)	619 (10.4%)		285 (8.7%)	80 (13%)	37 (14.2%)	25 (10.9%)	192 (11.9%)	
No HSD < 6.3%	17,520 (16%)	16,078 (19.3%)	1105 (5.5%)	337 (5.6%)		123 (3.8%)	41 (6.7%)	18 (6.9%)	8 (3.5%)	147 (9.1%)	
Missing	11,185 (10.2%)	8780 (10.5%)	1951 (9.7%)	454 (7.6%)		271 (8.3%)	43 (7%)	14 (5.4%)	16 (7%)	110 (6.8%)	
Neighborhood income											
<$40,227	23,505 (21.5%)	14,067 (16.8%)	8954 (44.7%)	1711 (28.6%)	<0.001	975 (29.9%)	237 (38.5%)	72 (27.6%)	80 (34.9%)	347 (21.5%)	<0.001
$40,227–$50,353	25,164 (23%)	23,526 (28.2%)	2581 (12.9%)	1174 (19.6%)		511 (15.7%)	105 (17.1%)	40 (15.3%)	41 (17.9%)	477 (29.6%)	
$50,354–$63,332	25,675 (23.4%)	18,810 (22.5%)	3911 (19.5%)	1285 (21.5%)		741 (22.7%)	118 (19.2%)	72 (27.6%)	53 (23.1%)	301 (18.7%)	
≥$63,333	24,821 (22.7%)	18,167 (21.8%)	2605 (13%)	1354 (22.6%)		764 (23.4%)	112 (18.2%)	63 (24.1%)	39 (17%)	376 (23.3%)	
Missing	10,337 (9.4%)	8927 (10.7%)	1975 (9.9%)	455 (7.6%)		272 (8.3%)	43 (7%)	14 (5.4%)	16 (7%)	110 (6.8%)	
Insurance status											
No	8082 (7.4%)	5027 (6%)	1923 (9.6%)	1132 (18.9%)	<0.001	635 (19.5%)	43 (7%)	21 (8%)	18 (7.9%)	415 (25.8%)	<0.001
Other government	1239 (1.1%)	1011 (1.2%)	186 (0.9%)	42 (0.7%)		25 (0.8%)	5 (0.8%)	2 (0.8%)	1 (0.4%)	9 (0.6%)	
Medicaid	23,566 (21.5%)	15,223 (18.2%)	6032 (30.1%)	2311 (38.7%)		1376 (42.2%)	230 (37.4%)	68 (26.1%)	104 (45.4%)	533 (33.1%)	
Medicare	20,502 (18.7%)	15,517 (18.6%)	4193 (20.9%)	792 (13.2%)		375 (11.5%)	138 (22.4%)	60 (23%)	43 (18.8%)	176 (10.9%)	
Private	53,673 (49%)	45,038 (53.9%)	7123 (35.6%)	1512 (25.3%)		723 (22.2%)	189 (30.7%)	108 (41.4%)	61 (26.6%)	431 (26.8%)	
Unknown	2440 (2.2%)	1681 (2%)	569 (2.8%)	190 (3.2%)		129 (4%)	10 (1.6%)	2 (0.8%)	2 (0.9%)	47 (2.9%)	
Rurality and urban influence											
Rural	1857 (1.7%)	1647 (2%)	194 (1%)	16 (0.3%)	<0.001	15 (0.5%)	0 (0%)	0 (0%)	0 (0%)	1 (0.1%)	<0.001
Urban	16,122 (14.7%)	14,216 (17%)	1723 (8.6%)	183 (3.1%)		144 (4.4%)	11 (1.8%)	5 (1.9%)	3 (1.3%)	20 (1.2%)	
Metro	87,819 (80.2%)	64,414 (77.1%)	17,702 (88.4%)	5703 (95.4%)		3067 (94%)	596 (96.9%)	255 (97.7%)	222 (96.9%)	1563 (97%)	
Missing	3704 (3.4%)	3220 (3.9%)	407 (2%)	77 (1.3%)		37 (1.1%)	8 (1.3%)	1 (0.4%)	4 (1.7%)	27 (1.7%)	
Charlson–Deyo index											
0	92,860 (84.8%)	71,540 (85.7%)	16,060 (80.2%)	5260 (88%)	<0.001	2896 (88.8%)	483 (78.5%)	230 (88.1%)	194 (84.7%)	1457 (90.4%)	<0.001
1	12,358 (11.3%)	9047 (10.8%)	2750 (13.7%)	561 (9.4%)		297 (9.1%)	89 (14.5%)	27 (10.3%)	25 (10.9%)	123 (7.6%)	
2	2765 (2.5%)	2019 (2.4%)	643 (3.2%)	103 (1.7%)		46 (1.4%)	28 (4.6%)	4 (1.5%)	6 (2.6%)	19 (1.2%)	
3+	1519 (1.4%)	891 (1.1%)	573 (2.9%)	55 (0.9%)		24 (0.7%)	15 (2.4%)	0 (0%)	4 (1.7%)	12 (0.7%)	
Facility type											
Community cancer program	3814 (3.5%)	3077 (3.7%)	499 (2.5%)	238 (4%)	<0.001	165 (5.1%)	25 (4.1%)	3 (1.1%)	7 (3.1%)	38 (2.4%)	<0.001
Comprehensive community cancer program	27,412 (25%)	22,431 (26.9%)	4109 (20.5%)	872 (14.6%)		479 (14.7%)	88 (14.3%)	64 (24.5%)	42 (18.3%)	199 (12.4%)	
Academic/research program	34,775 (31.8%)	23,957 (28.7%)	8166 (40.8%)	2652 (44.4%)		1467 (45%)	272 (44.2%)	74 (28.4%)	120 (52.4%)	719 (44.6%)	
Integrated network cancer program	15,302 (14%)	11,871 (14.2%)	2804 (14%)	627 (10.5%)		265 (8.1%)	62 (10.1%)	71 (27.2%)	22 (9.6%)	207 (12.8%)	
Missing	28,199 (25.8%)	22,161 (26.5%)	4448 (22.2%)	1590 (26.6%)		887 (27.2%)	168 (27.3%)	49 (18.8%)	38 (16.6%)	448 (27.8%)	
Facility location											
Northeast	15,878 (14.5%)	11,846 (14.2%)	3104 (15.5%)	928 (15.5%)	<0.001	81 (2.5%)	297 (48.3%)	31 (11.9%)	158 (69%)	361 (22.4%)	<0.001
South	33,451 (30.5%)	23,692 (28.4%)	8723 (43.6%)	1036 (17.3%)		444 (13.6%)	108 (17.6%)	166 (63.6%)	29 (12.7%)	289 (17.9%)	
Midwest	19,605 (17.9%)	16,358 (19.6%)	2970 (14.8%)	277 (4.6%)		197 (6%)	30 (4.9%)	7 (2.7%)	3 (1.3%)	40 (2.5%)	
West	12,369 (11.3%)	9440 (11.3%)	781 (3.9%)	2148 (35.9%)		1654 (50.7%)	12 (2%)	8 (3.1%)	1 (0.4%)	473 (29.4%)	
Missing	28,199 (25.8%)	22,161 (26.5%)	4448 (22.2%)	1590 (26.6%)		887 (27.2%)	168 (27.3%)	49 (18.8%)	38 (16.6%)	448 (27.8%)	
Stage											
I	50,542 (46.2%)	40,112 (48%)	7745 (38.7%)	2685 (44.9%)	<0.001	1470 (45.1%)	237 (38.5%)	105 (40.2%)	94 (41%)	779 (48.4%)	0.002
II	18,454 (16.9%)	13,555 (16.2%)	3738 (18.7%)	1161 (19.4%)		640 (19.6%)	132 (21.5%)	42 (16.1%)	44 (19.2%)	303 (18.8%)	
III	24,017 (21.9%)	17,577 (21.1%)	5055 (25.2%)	1385 (23.2%)		735 (22.5%)	163 (26.5%)	79 (30.3%)	64 (27.9%)	344 (21.4%)	
IV	16,489 (15.1%)	12,253 (14.7%)	3488 (17.4%)	748 (12.5%)		418 (12.8%)	83 (13.5%)	35 (13.4%)	27 (11.8%)	185 (11.5%)	
Tumor grade											
I	9573 (8.7%)	8004 (9.6%)	1078 (5.4%)	491 (8.2%)	<0.001	272 (8.3%)	52 (8.5%)	19 (7.3%)	15 (6.6%)	133 (8.3%)	0.91
II	30,667 (28%)	23,520 (28.2%)	5482 (27.4%)	1665 (27.8%)		918 (28.1%)	168 (27.3%)	66 (25.3%)	64 (27.9%)	449 (27.9%)	
III	28,083 (25.6%)	20,864 (25%)	5650 (28.2%)	1569 (26.2%)		877 (26.9%)	156 (25.4%)	73 (28%)	60 (26.2%)	403 (25%)	
Undifferentiated	1499 (1.4%)	1145 (1.4%)	248 (1.2%)	106 (1.8%)		62 (1.9%)	6 (1%)	5 (1.9%)	2 (0.9%)	31 (1.9%)	
Missing	39,680 (36.2%)	29,964 (35.9%)	7568 (37.8%)	2148 (35.9%)		1134 (34.8%)	233 (37.9%)	98 (37.5%)	88 (38.4%)	595 (36.9%)	
Tumor histology											
Adenocarcinoma	25,073 (22.9%)	21,250 (25.5%)	2557 (12.8%)	1266 (21.2%)	<0.001	696 (21.3%)	100 (16.3%)	52 (19.9%)	52 (22.7%)	366 (22.7%)	0.03
Sarcoma	465 (0.4%)	6 (0%)	1 (0%)	0 (0%)		0 (0%)	0 (0%)	0 (0%)	0 (0%)	0 (0%)	
Squamous cell carcinoma	7 (0%)	54,679 (65.5%)	15,605 (77.9%)	4162 (69.6%)		2268 (69.5%)	458 (74.5%)	191 (73.2%)	154 (67.2%)	1091 (67.7%)	
Unspecified carcinoma	74,446 (68%)	2022 (2.4%)	590 (2.9%)	137 (2.3%)		67 (2.1%)	18 (2.9%)	6 (2.3%)	7 (3.1%)	39 (2.4%)	
Other	6762 (6.2%)	352 (0.4%)	96 (0.5%)	17 (0.3%)		10 (0.3%)	4 (0.7%)	2 (0.8%)	0 (0%)	1 (0.1%)	
Unknown	2749 (2.5%)	5188 (6.2%)	1177 (5.9%)	397 (6.6%)		222 (6.8%)	35 (5.7%)	10 (3.8%)	16 (7%)	114 (7.1%)	

The Hispanic/Latinx cohort had a mean age of 49.7 years which was younger than the mean age for both the NHW and NHB cohorts (50.2 and 52.0 years, respectively; *p* < 0.001). Hispanic/Latinx patients were more likely to live in neighborhoods with the highest percentage of individuals who did not graduate high school (59.8%) compared to NHW (18.6%) and NHB patients (42.3%) (*p* < 0.001). However, a larger proportion of NHB (44.7%) individuals lived in neighborhoods with the lowest median incomes compared to NHW (16.9%) and Hispanic/Latinx groups (28.6%) (*p* < 0.001). The Hispanic/Latinx group had the highest percentage of uninsured patients (18.9%) when compared to NHB (9.6%) and NHW (6.0%) patients (*p* < 0.001). Most of the Hispanic/Latinx group received care at an academic/research center (44.4%) and lived in a metropolitan area (95.4%). The racial and ethnic distribution of patients varied by geographic region. Most Hispanic/Latinx patients were treated in facilities located in the Western region of the U.S. (35.9%), whereas most NHW and NHB patients were treated in facilities located in the South (28.4% and 43.6%, respectively; *p* < 0.001, Table 1).

Among Hispanic/Latinx patients, average age ranged from 49.3 to 54.5 years. More Mexican (64.9%) and Dominican (62.9%) individuals lived in neighborhoods with the highest percentage of individuals who did not graduate high school compared to CSA (54.1%), Cuban (51.0%), and PR (50.2%) groups (*p* < 0.001). The PR group had the highest proportion of patients (38.5%) living in the lowest‐income neighborhoods, compared to Dominican (34.9%), Mexican (29.9%), Cuban (27.6%), and CSA (21.5%) groups (*p* < 0.001). The CSA group had the highest percentage (25.8%) of uninsured individuals, and the Mexican group had the lowest proportion (22.2%) of privately insured individuals compared to other Hispanic/Latinx subpopulations (*p* < 0.001). Conversely, the PR group had the lowest percentage (7.0%) of uninsured patients, and the Cuban group had the highest percentage (41.4%) of privately insured patients (*p* < 0.001). Healthcare facility location varied by subgroup, with 50.7% of the Mexican population located in the geographic West, 48.3% of the PR population in the Northeast, 63.6% of the Cuban population in the South, and 69.0% of the Dominican population in the Northeast. Within the CSA group, 29.4% were located in the West, 22.4% in the Northeast, and 18.9% in the South (Table 1).

### 3.2 Tumor characteristics and stage

**FIGURE 1:**
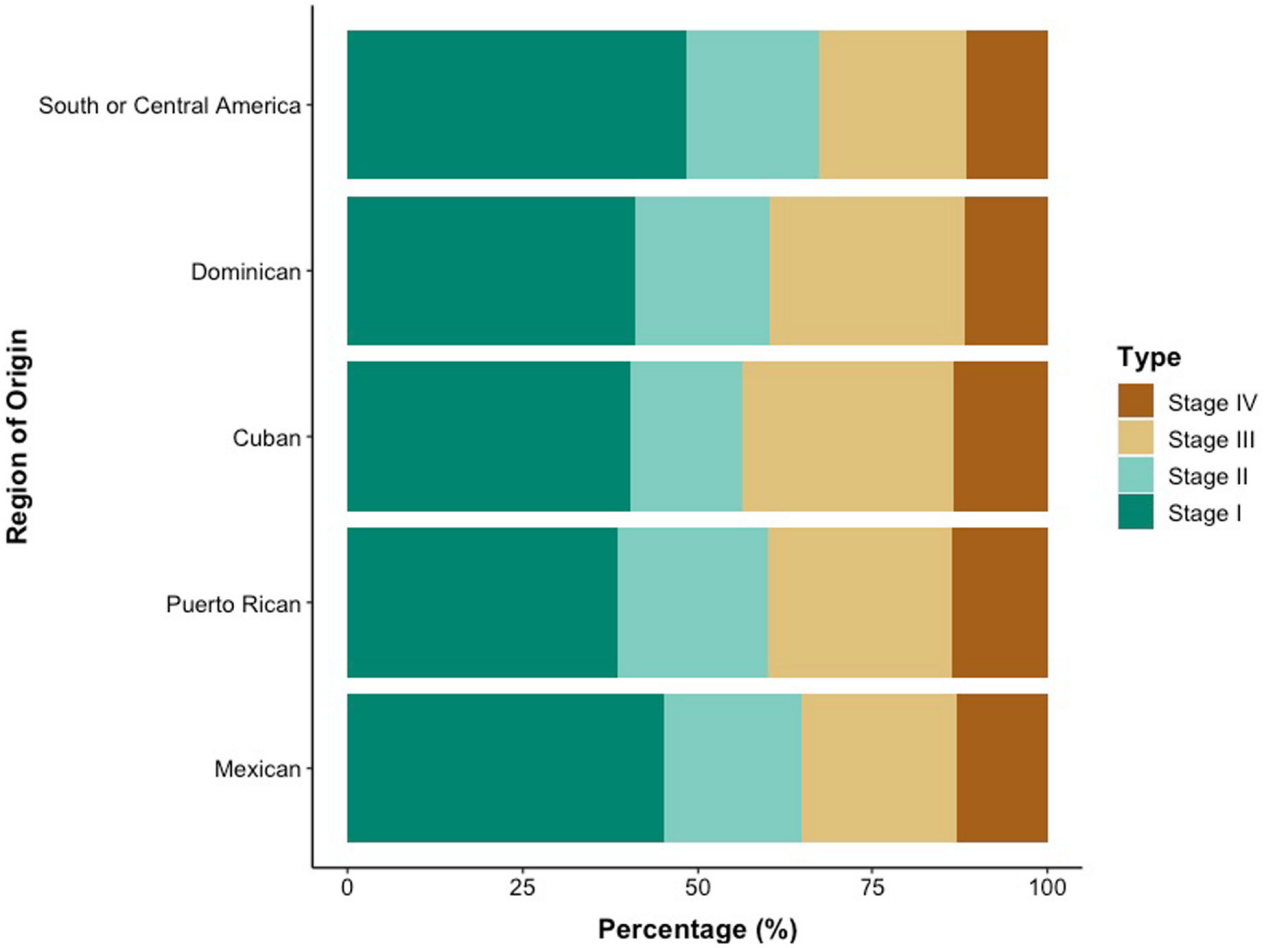
Cervical cancer stage at presentation by Hispanic/Latinx subpopulation.

Among Hispanic/Latinx subpopulations, the PR group had the lowest percentage of patients with localized disease (38.5%) and the highest percentage (13.5%) of patients with distant disease. The Cuban subpopulation had the second highest percentage (13.4%) of patients with distant disease (Figure 1, Table 1). When adjusting for age, education, neighborhood income, and insurance status, PR patients were 39.0% more likely than Mexican patients (reference group) to present with regional versus localized disease (OR 1.39; 95% CI, 1.14–1.68). When additionally adjusting for tumor grade, histology, Charlson–Deyo Comorbidity Index, year of diagnosis, facility location, and facility type, PR patients were 49.0% more likely than Mexican patients to present with regional versus localized disease (OR 1.49; 95% CI, 1.27–1.76) and 48.0% more likely to present with distant versus localized disease (OR 1.48; 95% CI 1.34–1.64). In this secondary adjusted model, Cuban patients were also 21.0% more likely than Mexican patients to present with regional versus localized disease (OR 1.21; 95% CI, 1.15–1.28) and 11.0% more likely to present with distant versus localized (OR 1.11; 95% CI, 1.07–1.15). No significant differences in odds of presenting with later‐stage disease at diagnosis were found among other Hispanic/Latinx subpopulations (Table 2).

**TABLE 2:** Comparison in stage at presentation among Hispanic/Latinx subpopulations.

Covariate	Odds ratio with 95% confidence interval		
	Adjusted model 1	Adjusted model 2	Adjusted model 3
Hispanic subgroup			
Stage II–III vs. Stage I			
Mexican^a^	1.00	1.00	1.00
Cuban	1.07, (0.81, 1.41)	1.17, (0.88, 1.55)	1.21, (1.15, 1.28)
Puerto Rican	1.3, (1.07, 1.57)	1.39, (1.14, 1.68)	1.49, (1.27, 1.76)
Central or South American	0.88, (0.77, 1)	0.88, (0.78, 1.01)	0.85, (0.74, 0.97)
Dominican	1.05, (0.79, 1.41)	1.09, (0.81, 1.46)	1.09, (1, 1.18)
Stage IV vs. Stage I			
Mexican^a^	1.00	1.00	1.00
Cuban	0.93, (0.62, 1.39)	0.99, (0.66, 1.51)	1.11, (1.07, 1.15)
Puerto Rican	1.17, (0.88, 1.55)	1.21, (0.91, 1.62)	1.48, (1.34, 1.64)
Central or South American	0.81, (0.67, 0.99)	0.81, (0.66, 0.99)	0.78, (0.67, 0.92)
Dominican	0.78, (0.5, 1.23)	0.81, (0.51, 1.27)	0.8, (0.76, 0.83)

*Note*: Model 1 was adjusted for age. Model 2 was adjusted for age, education, neighborhood income, and insurance status. Model 3 was adjusted for age, education, neighborhood income, insurance status, tumor grade, tumor histology, Charlson–Deyo index, year of diagnosis, facility location and facility type.

^a^ The element was the reference level for a variable.

### 3.3 Associated factors

Age, insurance status, neighborhood income, and education level were associated with cervical cancer stage at diagnosis among Hispanic/Latinx subpopulations (Table 3). For every year gained in age, the odds of presenting with more advanced‐stage disease increased by 3.0% for regional versus localized (OR 1.03; 95% CI, 1.02–1.04) and by 4% for distant versus localized (OR 1.04; 95% CI, 1.04–1.05). Uninsured Hispanic/Latinx patients had 37.0% higher odds of presenting with regional versus localized disease (OR 1.37; 95% CI, 1.19–1.58) and 47.0% higher odds of presenting with distant versus localized disease than insured patients (OR 1.47; 95% CI, 1.33–1.62). Hispanic/Latinx patients with Medicaid had 21.0% higher odds of presenting with regional versus localized disease (OR 1.21; 95% CI, 1.06–1.39) and 58.0% higher odds of presenting with distant versus localized disease (OR 1.58; 95% CI, 1.37–1.83) as compared to insured patients. When compared to a median neighborhood income level of under $40,227, those within the $40,227–$50,353 category were more likely to present with distant versus localized disease (OR 1.19; 95% CI, 1.01–1.39). There was otherwise no significant association between median neighborhood income and stage at presentation. When compared to a neighborhood high school diploma rate of >17.6%, all other categories with lower education levels were associated with a significantly higher odds of presenting with distant versus localized disease (Table 3).

**TABLE 3:** Factors associated with stage at diagnosis among Hispanic/Latinx patients.

Covariate	Odds Ratio with 95% Confidence Interval					
	Adjusted Model 1c	Adjusted Model 1a	Adjusted Model 2b	Adjusted Model 1c	Adjusted Model 1a	Adjusted Model 2b
	Stage II–III vs. Stage I			Stage IV vs. Stage I		
Age	1.03, (1.03, 1.03)	1.03, (1.03, 1.04)	1.03, (1.02, 1.04)	1.05, (1.04, 1.05)	1.05, (1.04, 1.06)	1.04, (1.04, 1.05)
Education (No high school diploma)						
≥17.6%^a^	^b^	1.00	1.00	^b^	1.00	1.00
<6.3%	^b^	0.84, (0.63, 1.13)	0.9, (0.83, 0.98)	^b^	1.17, (0.77, 1.77)	1.22, (1.15, 1.29)
6.3%–10.8%	^b^	0.92, (0.73, 1.14)	1.07, (0.92, 1.24)	^b^	1.27, (0.92, 1.74)	1.41, (1.29, 1.55)
10.9%–17.5%	^b^	1.02, (0.86, 1.2)	1.05, (0.91, 1.21)	^b^	1.03, (0.8, 1.33)	1.15, (1.06, 1.25)
Unknown	^b^	^c^	1.01, (0.93, 1.11)	^b^	3.37, (2.86, 3.96)	1.08, (1, 1.16)
Neighborhood income						
<$40,227^a^	^b^	1.00	1.00	^b^	1.00	1.00
$40,227–$50,353	^b^	1.00, (0.85, 1.17)	1.05, (0.92, 1.2)	^b^	1.12, (0.88, 1.44)	1.19, (1.01, 1.39)
$50,354–$63,332	^b^	0.99, (0.84, 1.17)	0.99, (0.87, 1.13)	^b^	1.09, (0.85, 1.4)	1.09, (0.92, 1.28)
≥$63,333	^b^	1.05, (0.85, 1.29)	0.98, (0.87, 1.1)	^b^	1.16, (0.85, 1.58)	1.07, (0.93, 1.24)
Unknown	^b^	^c^	1.01, (0.93, 1.11)	^b^	0.41, (0.35, 0.49)	1.08, (1, 1.16)
Insurance status						
Private^a^	^b^	1.00	1.00	^b^	1.00	1.00
No	^b^	1.45, (1.23, 1.72)	1.37, (1.19, 1.58)	^b^	1.48, (1.13, 1.95)	1.47, (1.33, 1.62)
Medicaid	^b^	1.3, (1.13, 1.51)	1.21, (1.06, 1.39)	^b^	1.57, (1.25, 1.97)	1.58, (1.37, 1.83)
Medicare	^b^	1.03, (0.83, 1.27)	0.96, (0.86, 1.08)	^b^	1.32, (0.98, 1.79)	1.31, (1.17, 1.46)
Other	^b^	2.11, (1.09, 4.08)	2.58, (2.57, 2.6)	^b^	1.14, (0.32, 4)	1.82, (1.82, 1.83)
Unknown	^b^	1.21, (0.87, 1.69)	1.1, (1.05, 1.15)	^b^	1.66, (1.02, 2.7)	1.67, (1.64, 1.71)
Tumor grade						
I^a^	^b^	1.00	1.00	^b^	1.00	1.00
II	^b^	^b^	2.43, (2.2, 2.69)	^b^	^b^	1.76, (1.52, 2.03)
III	^b^	^b^	3.81, (3.44, 4.22)	^b^	^b^	3.58, (3.11, 4.12)
Undifferentiated	^b^	^b^	3.17, (3.15, 3.19)	^b^	^b^	3.03, (3.01, 3.05)
Unknown	^b^	^b^	2.63, (2.39, 2.9)	^b^	^b^	2.58, (2.26, 2.95)
Tumor histology						
Squamous cell carcinoma^a^	^b^	1.00	1.00	^b^	1.00	1.00
Adenocarcinoma	^b^	^b^	0.44, (0.38, 0.51)	^b^	^b^	0.68, (0.62, 0.75)
Unspecified carcinoma	^b^	^b^	1.24, (1.21, 1.26)	^b^	^b^	2.6, (2.55, 2.65)
Other	^b^	^b^	4.13, (4.12, 4.14)	^b^	^b^	7.98, (7.96, 8.01)
Unknown	^b^	^b^	0.68, (0.56, 0.82)	^b^	^b^	1.12, (1, 1.26)
Charlson–Deyo index						
0^a^	^b^	1.00	1.00	^b^	1.00	1.00
1	^b^	^b^	0.95, (0.81, 1.12)	^b^	^b^	0.9, (0.83, 0.98)
2	^b^	^b^	0.4, (0.4, 0.41)	^b^	^b^	0.54, (0.53, 0.54)
3+	^b^	^b^	0.8, (0.79, 0.8)	^b^	^b^	1.2, (1.2, 1.21)
Facility location						
Midwest^a^	^b^	1.00	1.00	^b^	1.00	1.00
Northeast	^b^	^b^	1.09, (0.98, 1.2)	^b^	^b^	0.99, (0.87, 1.12)
South	^b^	^b^	0.95, (0.85, 1.05)	^b^	^b^	0.91, (0.81, 1.03)
West	^b^	^b^	1.03, (0.94, 1.14)	^b^	^b^	1.23, (1.09, 1.4)
Facility type						
Comprehensive community cancer program^a^	^b^	1.00	1.00	^b^	1.00	1.00
Academic/research program	^b^	^b^	1.02, (0.9, 1.16)	^b^	^b^	0.91, (0.78, 1.06)
Community cancer program	^b^	^b^	0.85, (0.78, 0.92)	^b^	^b^	0.85, (0.81, 0.89)
Integrated network cancer program	^b^	^b^	1.06, (0.91, 1.23)	^b^	^b^	1.02, (0.92, 1.12)
Unknown	^b^	^b^	1, (1)	^b^	^b^	1, (1)
Year of Diagnosis	^b^	^b^	1.02, (1.02, 1.02)	^b^	^b^	1.06, (1.06, 1.06)

*Note*: The model was adjusted for age. The model was adjusted for age, education, neighborhood income, and insurance status. The model was adjusted for age, education, neighborhood income, insurance status, tumor grade, tumor histology, Charlson–Deyo Index, year of diagnosis, facility location and facility type.

^a^ The element was the reference level for a variable.

^b^ The element was not available in a model.

^c^ The estimate was invalid due to insufficient observations.

## 4 DISCUSSION

To our knowledge, this is one of the first studies to present disaggregated data for Hispanic/Latinx subpopulations as it pertains to cervical cancer stage. The above findings demonstrate an association between insurance status, education level, and neighborhood income with distant stage presentation at diagnosis among patients of Hispanic/Latinx subpopulations. It was noted PR and Cuban groups had higher likelihood of advanced stage at presentation, even after controlling for variation in socioeconomic characteristics. Importantly, insurance status was a major predictor of stage at presentation, providing direct evidence that a patient's health insurance coverage (or lack thereof) has a significant impact on delaying cancer care. Not only does this study illustrate the importance of disaggregated data, but also provides a framework for further study and needed resource allocation to structural factors that impact health outcomes.

Our findings show that Cuban and PR subpopulations have more advanced stage at presentation compared to Mexican patients. These findings are aligned with current literature demonstrating that these populations have incidence rates of major cancers and higher cancer mortality than other Hispanic/Latinx groups.^11^, ^16^, ^17^, ^18^ In a study of Hispanic/Latinx cervical cancer patients in Florida, PR patients had a mortality rate ratio of 1.34, higher than the total Hispanic/Latinx cohort (0.97).^8^ The causes of these disparities in cancer outcomes among all Hispanic/Latinx subpopulations are attributed to the intersection of numerous socioeconomic, structural, and behavioral factors with no apparent single explanation.^15^, ^16^ For example, limited access to healthcare resources among Hispanic/Latinx populations in the U.S. is associated with inadequate cancer screening and lower rates of HPV vaccination.^15^, ^16^, ^18^, ^19^, ^20^ High rates of cervical cancer among PR populations have also been associated with factors such as higher prevalence of HPV and increased burden of HIV.^19^ Additionally, smoking is strongly associated with cervical cancer incidence rates and is documented to be more prevalent among certain Hispanic/Latinx communities, especially Cuban and PR populations, which may contribute to higher rates of advanced cancer.^8^, ^10^, ^16^

Around 24% of Hispanic/Latinx individuals in the U.S. live below the poverty line, and an estimated 35% have less than a high school education, which have both been associated with higher rates of infection‐related cancer such as cervical cancer.^8^ We found an association between neighborhood education level and stage at presentation, but no such association with neighborhood income level. Income at the individual and national level, as well as education, have been associated with lower cervical cancer screening rates and worse outcomes in prior studies conducted in various countries.^4^, ^21^, ^22^ In the U.S., NHB populations living in high‐poverty areas (defined as >20% poverty) were found to be 1.2 times less likely to have had routine cervical cancer screening when compared to those living in <5% poverty, even after adjusting for other potential causes.^22^ However, these studies evaluated primarily individual‐level or national‐level income, which are different measures than neighborhood income. Other analyses have demonstrated close association between neighborhood‐level socioeconomic disparities and cervical cancer stage, yet data on neighborhood‐level income and cervical cancer outcomes are limited.^4^ The lack of significant association in our findings between neighborhood income level and stage at presentation may be attributed to a true correlation. However, differences in cost of living and local or state resources allocated specifically to cervical cancer may also account for this discrepancy.

Previous studies have also highlighted the association between insurance status and cervical cancer outcomes.^23^, ^24^, ^25^, ^26^ Patients without insurance or with public insurance are less likely to obtain recommended cervical cancer screening, which may contribute increased rates of cervical cancer mortality.^24^, ^25^ Patients with public insurance are also more likely to present with distant stage cervical cancer when compared to those with private insurance.^25^, ^26^ Publicly insured and uninsured status were found to be predictors of distant stage at presentation in our multivariable‐adjusted logistic regression model. The total Hispanic/Latinx cohort was more likely to be uninsured overall, with differences in rate of insurance among each subpopulation.

Notably, the insurance status of the PR subpopulation more closely resembled that of the NHW group than the total Hispanic/Latinx cohort, indicating that this may not have been a primary mediator of distant stage presentation in these patients. Rather, some studies have indicated that PR populations may suffer from psychosocial burdens secondary to PR's status as U.S. territory and devastation from recent natural disasters including Hurricane Maria, thus contributing to poor health outcomes.^27^, ^28^

In addition to the above, we must also consider systemic racism as a potential cause of the presented findings. Structural racism results in systematic disinvestment and shapes healthcare access and quality among communities of color, leading to poorer outcomes.^29^ For example, Hispanic/Latinx ovarian and endometrial cancer patients have been shown to be less likely to receive standard of care treatment when controlled for other factors.^17^ This suggests that at least some of the disparities in care based on race and/or ethnicity are likely a product of the structures of systemic inequities built into our healthcare system. While measuring these factors can be difficult when using administrative data sets, we acknowledge that these elements potentially contributed to distant stage at presentation in Hispanic/Latinx populations in our study.

Strengths of the current study include the large‐scale and consistent NCDB data on Hispanic/Latinx subpopulations that fills a critical research gap. However, our study also has limitations. First, the NCDB relies on hospital claims data; therefore, key demographic information may not always have been accurately self‐reported by patients and may have been incorrectly reported.^14^ Second, unknown nationality among Hispanic/Latinx resulted in incomplete demographic information, which also further limited our ability to analyze additional Hispanic/Latinx groups based on country of origin due to unknown nationality. Similarly, because our data were already categorized by NCDB criteria, combining CSA patients into one group limits our ability to completely disaggregate our study population and may mask some underlying differences. Lastly, we lack data on specific patient‐related factors that could have delayed presentation to care and caused later stage at presentation, such as citizenship status and other socioeconomic factors.

## 5 CONCLUSION

Racial disparities continue to result in excess morbidity and mortality within minoritized populations, and as such, we must continue to promote health equity.^30^ Hispanic/Latinx patients have historically been considered as a homogenous group in health outcomes research. Disaggregating health data are an important step in identifying and addressing barriers in access to care within specific subpopulations.^31^, ^32^ Our findings highlight the differences in cervical cancer stage at presentation among Hispanic/Latinx subpopulations and the heterogeneity of this group. We identified significant differences in income and education between Hispanic/Latinx subpopulations. Furthermore, we identified differences in cervical cancer stage at presentation among Hispanic/Latinx subpopulations and showed that insurance status is a major predictor of stage at presentation. Thus, further research into targeting these structural factors within specific Hispanic/Latinx subpopulations is warranted.

## AUTHOR CONTRIBUTIONS

**Andreea Ioana Dinicu:** Writing – original draft (lead); writing – review and editing (equal). **Shayan Dioun:** Writing – original draft (equal); writing – review and editing (equal). **Mandy Goldberg:** Conceptualization (equal); data curation (equal); methodology (equal); writing – review and editing (supporting). **Danielle M. Crookes:** Conceptualization (equal); methodology (equal); project administration (equal); writing – review and editing (supporting). **Yongzhe Wang:** Formal analysis (lead); writing – original draft (supporting); writing – review and editing (equal). **Ana I. Tergas:** Conceptualization (equal); data curation (equal); investigation (equal); methodology (equal); supervision (equal); writing – original draft (equal); writing – review and editing (equal).

## FUNDING INFORMATION

Dr. Tergas was supported by a career development award from NCI (K08 CA245193‐01).

## CONFLICT OF INTEREST STATEMENT

Dr. Tergas participated in an advisory board meeting for Merck. The other authors have no conflict of interest to declare.

## Data Availability

Data subject to third party restrictions.
